# Li_2_GeMo_3_O_8_: a novel reduced molybdenum oxide containing Mo_3_O_13_ cluster units

**DOI:** 10.1107/S2056989016009750

**Published:** 2016-06-21

**Authors:** Philippe Gall, Patrick Gougeon

**Affiliations:** a Institut des Sciences, Chimiques de Rennes, UMR 6226 CNRS – INSA Rennes – Université de Rennes 1, Avenue du Général Leclerc, 35042 Rennes Cedex, France

**Keywords:** crystal structure, reduced molybdenum oxide, triangular Mo_3_ cluster, lithium, germanium

## Abstract

The crystal structure of the title compound, dilithium germanium trimolybdenum octa­oxide, consists of distorted hexa­gonal-close-packed oxygen layers with stacking sequence *ABAC* along [001] that are held together by alternating lithium–germanium and molybdenum layers. The two Li^+^ and Ge^4+^ ions all have site symmetry 3*m*. and occupy, respectively, tetra­hedral and octa­hedral sites in the ratio 2:1. The Mo atom has a formal oxidation state of +3.3 and occupies an octa­hedral site (site symmetry .*m*.) and forms strongly bonded triangular cluster units [Mo—Mo distance = 2.4728 (8) Å] involving three MoO_6_ octa­hedra that are each shared along two edges, constituting an Mo_3_O_13_ unit.

## Abstract 2

The title compound crystallizes in the space group *P*6_3_ *mc* and is isotypic with Li_2_SnMo_3_O_8_ and Li_2_InMo_3_O_8._ Its crystal structure contains triangular Mo_3_ clusters units whereby the tetra­valent oxidation state of the germanium atom leads to 8 electrons per Mo_3_ cluster.

## Chemical context   

Reduced molybdenum oxides containing the Mo_3_O_13_ cluster unit crystallize either in the hexa­gonal space group type *P*6_3_ *mc* (*a* ∼ 5.7–5.8 Å, *c* ∼ 10.0–10.2 Å) or in the trigonal space group types *P*3*m*1 (*a* ∼ 5.7–5.8 Å, *c* ∼ 4.9–5.3 Å) or *R* 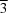 *m* (*a* ∼ 5.8–5.9 Å, *c* ∼ 30.0–30.1 Å). Representatives of the first family are the ternary compounds *M*_2_Mo_3_O_8_ (McCarroll *et al.*, 1957 ▸) where *M* is a divalent metal such as Mg, Zn, Fe, Co, Ni, Zn and Cd as well as the quaternary compounds ScZnMo_3_O_8_ and Li_2_ *M*Mo_3_O_8_ (*M* = Sn, In) (Gall *et al.*, 2013*a* ▸,*b* ▸). The Li*R*Mo_3_O_8_ series (*R* = Sc, Y, In, Sm, Gd, Tb, Dy, Ho, Er and Yb) (DeBenedittis & Katz, 1965 ▸; McCarroll, 1977 ▸) crystallize in the *P*3*m*1 space group and finally, LiZn_2_Mo_3_O_8_ and Zn_3_Mo_3_O_8_ (Torardi & McCarley, 1985 ▸) crystallize in space group *R* 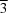 *m*. The crystal structures of all these compounds consist of distorted hexa­gonal-close-packed oxygen layers with stacking sequences *ABAC*, *ABAB* and *ABC* for compounds crystallizing in the space groups *P*6_3_ *mc*, *P*3*m*1 and *R* 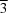 *m*, respectively. The oxygen layers are separated by alternating mixed-metal atom (Li, *M*, or *R*) layers and molybdenum layers. The metal atoms occupy both tetra­hedral and octa­hedral sites in a ratio of 1:1 (*M*_2_Mo_3_O_8_ and Li*R*Mo_3_O_8_) or 2:1 (LiZn_2_Mo_3_O_8_ and Zn_3_Mo_3_O_8_) between two adjacent oxygen layers. The molybdenum atoms occupy three quarters of the octa­hedral sites and form strongly bonded triangular cluster units involving three MoO_6_ octa­hedra that are each shared along two edges, the whole constituting an Mo_3_O_13_ unit. The Mo—Mo bonds within the trinuclear cluster units range from about 2.5 to 2.6 Å, and the number of electrons available for Mo–Mo bonding is six in *M*_2_Mo_3_O_8_ and Li*R*Mo_3_O_8_, seven in LiZn_2_Mo_3_O_8_ and Li_2_InMo_3_O_8_, and eight in Zn_3_Mo_3_O_8_ and Li_2_SnMo_3_O_8_. The energy-level diagram deduced from LCAO–MO calculations on the Mo_3_O_13_ unit shows three bonding orbitals (*a*1 and *e*), a non-bonding level (*a*1), and five anti-bonding orbitals (2*e* and *a*2) (Cotton, 1964 ▸). This explains why the compounds with seven electrons per Mo_3_ cluster unit are paramagnetic with moments corresponding to one unpaired electron per Mo_3_ cluster unit, and those with six and eight electrons per Mo_3_ show temperature-independent paramagnetism.

We present here the crystal structure of the new quaternary compound Li_2_GeMo_3_O_8_ in which the Mo_3_ cluster unit has eight electrons available for bonding.

## Structural commentary   

**Figure 1:**
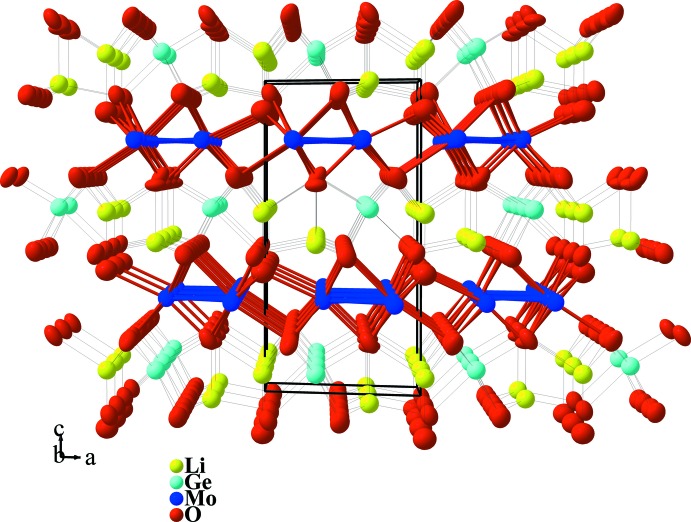
View of the crystal structure of Li_2_GeMo_3_O_8_ in a projection approximately along [010]. Displacement ellipsoids are drawn at the 97% probability level.

**Figure 2:**
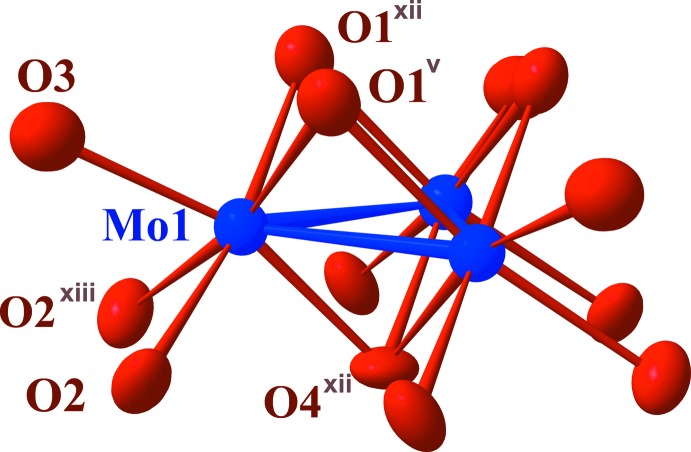
The Mo_3_O_13_ cluster unit with its numbering scheme, with ellipsoids drawn at the 97% probability level. [Symmetry codes: (v) *y* + 1, −*x* + *y*+2, *z* − 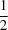; (xii) −*x* + 3, −*y* + 2, *z* − 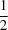; (xiii) −*y* + 2, *x*-*y* + 1, *z*; (xiv) −*x* + *y*+2, −*x* + 3, *z*.]

Li_2_GeMo_3_O_8_ is isotypic with the Li_2_ *M*Mo_3_O_8_ (*M* = Sn, In) compounds (Gall *et al.*, 2013*a* ▸,*b* ▸). Its crystal structure consists of distorted hexa­gonal-close-packed oxygen layers with stacking sequence *ABAC* along [001] that are held together by alternating lithium–germanium and molybdenum layers (Fig. 1 ▸). The Li^+^ and Ge^4+^ ions occupy, respectively, tetra­hedral and octa­hedral sites in the ratio 2:1. The Mo atoms occupy octa­hedral sites and form strongly bonded triangular cluster units involving three MoO_6_ octa­hedra that are each shared along two edges, constituting an Mo_3_O_13_ unit (Fig. 2 ▸). The Mo—Mo distance within the Mo_3_ triangle is 2.4728 (8) Å compared to 2.5036 (7) and 2.5455 (4) Å found in the tin and indium analogues, respectively. The Mo—O distances range from 2.004 (6) to 2.146 (3) Å (Table 1 ▸) while in Li_2_InMo_3_O_8_ they range from 2.0212 (17) to 2.1241 (16) Å and in Li_2_SnMo_3_O_8_ from 2.020 (6) to 2.122 (3) Å. The Li—O distances in the title structure range from 1.78 (2) to 2.012 (13) Å with average distances of 1.97 and 1.86 Å for the Li1 and Li2 sites, respectively. Both Li sites have site symmetry 3*m*.. For the Ge site, likewise with site symmetry 3*m*., the Ge—O distances are 3×1.883 (5) and 3×2.016 (5) Å. The average distance of 1.95 Å is close to the value of 1.92 Å calculated from the sum of the ionic radii of O^2−^ and Ge^4+^ in octa­hedral coordination according to Shannon & Prewitt (1969 ▸). The oxidation state of +4 for the Ge atoms was also confirmed from the Ge—O bond lengths by using the relationship of Brown & Wu (1976 ▸) {*s* = [*d*(Ge—O)/1.746]^−6.05^} which leads to a value of +3.5 (1). The latter relationship applied to Mo—O bonds {*s* = [*d*(Mo—O)/1.882]^−6^} yield an oxidation state of +3.38 for the Mo atom, and thus 7.86 electrons per Mo_3_ cluster unit, close to the expected value of 8. This is consistent with the chemical composition Li_2_^+^Ge^4+^Mo_3_^3.33+^O_8_^2−^.

## Database survey   

The *M*_2_Mo_3_O_8_ (Mg, Zn, Fe, Co, Ni, Zn and Cd) compounds containing triangular Mo_3_ clusters were first synthesized by McCarroll *et al.* (1957 ▸). They presented the results of a structure determination on Zn_2_Mo_3_O_8_ from photographic data (*R* = 0.118). Later, a refinement of the structure was accomplished by Ansell & Katz (1966 ▸) with an *R* factor of 0.069. Among the above compounds, it is inter­esting to note that Fe_2_Mo_3_O_8_ is a mineral known as kamiokite (Kanazawa & Sasaki, 1986 ▸). Later, DeBenedittis & Katz (1965 ▸) reported the existence of the Li*R*Mo_3_O_8_ (*R* = Sc and Y) compounds. Subsequently, McCarroll (1977 ▸) obtained isotypic compounds with *R* = In, Sm, Gd, Tb, Dy, Ho, Er, and Yb. In 1985, Torardi & McCarley (1985 ▸) described the new Mo_3_ cluster compounds LiZn_2_Mo_3_O_8_, Zn_3_Mo_3_O_8_ and ScZnMo_3_O_8_ and, in 2013, Gall *et al.* (2013*a* ▸,*b* ▸), the quaternary compounds Li_2_ *M*Mo_3_O_8_ (*M* = Sn and In).

## Synthesis and crystallization   

Single crystals of Li_2_GeMo_3_O_8_ were obtained by heating a mixture of Li_2_MoO_4_, O_2_, MoO_3_ and Mo with the nominal composition Li_2_GeMo_6_O_12_ at 1923 K for 72 h in a molybdenum crucible sealed under low argon pressure using an arc-welding system. The molybdate Li_2_MoO_4_ was synthesized by heating an equimolar ratio of MoO_3_ (CERAC 99.95%) and Li_2_CO_3_ (CERAC 99.9%) in an alumina vessel at 873 K in air over 12 h. Before use, the Mo powder was heated under a hydrogen flow at 1273 K for 6 h. The composition of the final crystals thus obtained was determined after a complete X-ray structural study on one of them.

## Refinement   

Crystal data, data collection and structure refinement details are summarized in Table 2 ▸. All atoms were refined with anisotropic displacement parameters, except for the Li atoms, which were refined isotropically.

## Supplementary Material

Crystal structure: contains datablock(s) I. DOI: 10.1107/S2056989016009750/wm5299sup1.cif

Structure factors: contains datablock(s) I. DOI: 10.1107/S2056989016009750/wm5299Isup2.hkl

CCDC reference: 1485831

Additional supporting information: crystallographic information; 3D view; checkCIF report

## Figures and Tables

**Table 1:** Selected bond lengths (Å)

Li1—O3	1.84 (2)	Ge1—O1^ix^	2.016 (5)
Li1—O2^i^	2.012 (13)	Ge1—O1^x^	2.016 (5)
Li1—O2^ii^	2.012 (13)	Ge1—O1^xi^	2.016 (4)
Li1—O2^iii^	2.012 (13)	Mo1—O4^xii^	2.004 (6)
Li2—O4	1.78 (3)	Mo1—O1^xii^	2.039 (4)
Li2—O1^iv^	1.892 (6)	Mo1—O1^v^	2.039 (4)
Li2—O1^v^	1.892 (6)	Mo1—O3	2.076 (3)
Li2—O1^vi^	1.892 (6)	Mo1—O2	2.146 (3)
Ge1—O2	1.883 (5)	Mo1—O2^xiii^	2.146 (3)
Ge1—O2^vii^	1.883 (5)	Mo1—Mo1^xiv^	2.4728 (8)
Ge1—O2^viii^	1.883 (5)		

Symmetry codes: (i) 
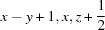
; (ii) 
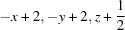
; (iii) 
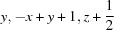
; (iv) 
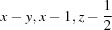
; (v) 
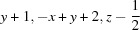
; (vi) 
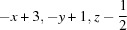
; (vii) 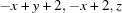; (viii) 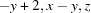; (ix) 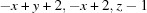; (x) 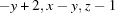; (xi) 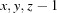; (xii) 
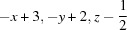
; (xiii) 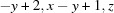; (xiv) 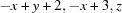.

**Table 2:** Experimental details

Crystal data	
Chemical formula	Li_2_GeMo_3_O_8_
*M*_r_	502.29
Crystal system, space group	Hexagonal, *P*6_3_ *m* *c*
Temperature (K)	293
*a*, *c* (Å)	5.7268 (3), 9.9841 (6)
*V* (Å^3^)	283.57 (3)
*Z*	2
Radiation type	Mo *K*α
μ (mm^−1^)	11.74
Crystal size (mm)	0.21 × 0.13 × 0.07

Data collection	
Diffractometer	Nonius KappaCCD
Absorption correction	Analytical (de Meulenaar & Tompa, 1965 ▸)
*T*_min_, *T*_max_	0.048, 0.157
No. of measured, independent and observed [*I* > 2σ(*I*)] reflections	4457, 522, 501
*R*_int_	0.063
(sin θ/λ)_max_ (Å^−1^)	0.807

Refinement	
*R*[*F* ^2^ > 2σ(*F* ^2^)], *wR*(*F* ^2^), *S*	0.030, 0.076, 1.10
No. of reflections	522
No. of parameters	31
No. of restraints	1
Δρ_max_, Δρ_min_ (e Å^−3^)	1.43, −1.33
Absolute structure	Flack (1983 ▸), 247 Friedel pairs
Absolute structure parameter	0.01 (3)

Computer programs: *COLLECT* (Nonius, 1998 ▸), *EVALCCD* (Duisenberg *et al.*, 2003 ▸), *SIR97* (Altomare *et al.*, 1999 ▸), *SHELXL97* (Sheldrick, 2008 ▸) and *DIAMOND* (Bergerhoff, 1996 ▸).

## supplementary crystallographic information

### Crystal data

**Table:**

Li_2_GeMo_3_O_8_	*D*_x_ = 5.883 Mg m^−^^3^
*M**_r_* = 502.29	Mo *K*α radiation, λ = 0.71073 Å
Hexagonal, *P*6_3_*m**c*	Cell parameters from 4457 reflections
Hall symbol: P 6c -2c	θ = 4.1–35.0°
*a* = 5.7268 (3) Å	µ = 11.74 mm^−^^1^
*c* = 9.9841 (6) Å	*T* = 293 K
*V* = 283.57 (3) Å^3^	Irregular block, black
*Z* = 2	0.21 × 0.13 × 0.07 mm
*F*(000) = 456	

### Data collection

**Table:**

Nonius KappaCCD diffractometer	522 independent reflections
Radiation source: fine-focus sealed tube	501 reflections with *I* > 2σ(*I*)
Graphite monochromator	*R*_int_ = 0.063
φ scans (κ = 0) + additional ω scans	θ_max_ = 35.0°, θ_min_ = 4.1°
Absorption correction: analytical (de Meulenaar & Tompa, 1965)	*h* = −7→9
*T*_min_ = 0.048, *T*_max_ = 0.157	*k* = −9→9
4457 measured reflections	*l* = −16→16

### Refinement

**Table:**

Refinement on *F*^2^	Primary atom site location: structure-invariant direct methods
Least-squares matrix: full	Secondary atom site location: difference Fourier map
*R*[*F*^2^ > 2σ(*F*^2^)] = 0.030	*w* = 1/[σ^2^(*F*_o_^2^) + (0.0515*P*)^2^ + 0.3716*P*] where *P* = (*F*_o_^2^ + 2*F*_c_^2^)/3
*wR*(*F*^2^) = 0.076	(Δ/σ)_max_ < 0.001
*S* = 1.10	Δρ_max_ = 1.43 e Å^−^^3^
522 reflections	Δρ_min_ = −1.33 e Å^−^^3^
31 parameters	Absolute structure: Flack (1983), 247 Friedel pairs
1 restraint	Absolute structure parameter: 0.01 (3)

### Special details

**Table:**

Geometry. All esds (except the esd in the dihedral angle between two l.s. planes) are estimated using the full covariance matrix. The cell esds are taken into account individually in the estimation of esds in distances, angles and torsion angles; correlations between esds in cell parameters are only used when they are defined by crystal symmetry. An approximate (isotropic) treatment of cell esds is used for estimating esds involving l.s. planes.
Refinement. Refinement of F^2^ against ALL reflections. The weighted R-factor wR and goodness of fit S are based on F^2^, conventional R-factors R are based on F, with F set to zero for negative F^2^. The threshold expression of F^2^ > 2sigma(F^2^) is used only for calculating R-factors(gt) etc. and is not relevant to the choice of reflections for refinement. R-factors based on F^2^ are statistically about twice as large as those based on F, and R- factors based on ALL data will be even larger.

### Fractional atomic coordinates and isotropic or equivalent isotropic displacement parameters (Å^2^)

**Table:**

	*x*	*y*	*z*	*U*_iso_*/*U*_eq_	
Li1	1.0000	1.0000	1.581 (2)	0.014 (3)*	
Li2	1.3333	0.6667	1.490 (2)	0.014 (3)*	
Ge1	1.3333	0.6667	1.09391 (9)	0.0076 (2)	
Mo1	1.37881 (9)	1.18940 (4)	1.30891 (9)	0.00660 (13)	
O1	1.4795 (4)	0.5205 (4)	1.9536 (5)	0.0081 (8)	
O2	1.1704 (6)	0.8296 (6)	1.1906 (5)	0.0081 (8)	
O3	1.0000	1.0000	1.3974 (7)	0.0110 (14)	
O4	1.3333	0.6667	1.6680 (8)	0.0080 (13)	

### Atomic displacement parameters (Å^2^)

**Table:**

	*U*^11^	*U*^22^	*U*^33^	*U*^12^	*U*^13^	*U*^23^
Ge1	0.0076 (3)	0.0076 (3)	0.0076 (4)	0.00381 (14)	0.000	0.000
Mo1	0.00622 (19)	0.00662 (16)	0.00683 (19)	0.00311 (10)	−0.00015 (18)	−0.00007 (9)
O1	0.0079 (13)	0.0079 (13)	0.0094 (18)	0.0045 (14)	0.0007 (8)	−0.0007 (8)
O2	0.0071 (12)	0.0071 (12)	0.010 (2)	0.0034 (13)	0.0017 (9)	−0.0017 (9)
O3	0.012 (2)	0.012 (2)	0.010 (3)	0.0058 (10)	0.000	0.000
O4	0.0102 (19)	0.0102 (19)	0.004 (3)	0.0051 (9)	0.000	0.000

### Geometric parameters (Å, º)

**Table:**

Li1—O3	1.84 (2)	Ge1—O1^xv^	2.016 (5)
Li1—O2^i^	2.012 (13)	Ge1—O1^xvi^	2.016 (4)
Li1—O2^ii^	2.012 (13)	Mo1—O4^xvii^	2.004 (6)
Li1—O2^iii^	2.012 (13)	Mo1—O1^xvii^	2.039 (4)
Li1—Mo1^i^	2.949 (17)	Mo1—O1^viii^	2.039 (4)
Li1—Mo1^iii^	2.949 (17)	Mo1—O3	2.076 (3)
Li1—Mo1^ii^	2.949 (17)	Mo1—O2	2.146 (3)
Li1—Mo1^iv^	3.305 (18)	Mo1—O2^v^	2.146 (3)
Li1—Mo1^v^	3.305 (18)	Mo1—Mo1^xviii^	2.4728 (8)
Li1—Mo1	3.305 (18)	Mo1—Mo1^xix^	2.4728 (8)
Li1—Li2	3.430 (9)	Mo1—Li1^xx^	2.949 (17)
Li1—Li2^vi^	3.430 (9)	O1—Li2^xxi^	1.892 (6)
Li2—O4	1.78 (3)	O1—Ge1^xxii^	2.016 (4)
Li2—O1^vii^	1.892 (6)	O1—Mo1^xxiii^	2.039 (4)
Li2—O1^viii^	1.892 (6)	O1—Mo1^xxiv^	2.039 (4)
Li2—O1^ix^	1.892 (6)	O2—Li1^xx^	2.012 (13)
Li2—Li1^x^	3.430 (9)	O2—Mo1^iv^	2.146 (3)
Li2—Li1^xi^	3.430 (9)	O3—Mo1^iv^	2.076 (3)
Ge1—O2	1.883 (5)	O3—Mo1^v^	2.076 (3)
Ge1—O2^xii^	1.883 (5)	O4—Mo1^iii^	2.004 (5)
Ge1—O2^xiii^	1.883 (5)	O4—Mo1^xxiv^	2.004 (5)
Ge1—O1^xiv^	2.016 (5)	O4—Mo1^xxiii^	2.004 (5)
			
O3—Li1—O2^i^	122.9 (5)	Li1^x^—Li2—Li1^xi^	113.2 (5)
O3—Li1—O2^ii^	122.9 (5)	O2—Ge1—O2^xii^	96.1 (2)
O2^i^—Li1—O2^ii^	93.3 (7)	O2—Ge1—O2^xiii^	96.1 (2)
O3—Li1—O2^iii^	122.9 (5)	O2^xii^—Ge1—O2^xiii^	96.1 (2)
O2^i^—Li1—O2^iii^	93.3 (7)	O2—Ge1—O1^xiv^	92.73 (17)
O2^ii^—Li1—O2^iii^	93.3 (7)	O2^xii^—Ge1—O1^xiv^	166.8 (2)
O3—Li1—Mo1^i^	140.4 (3)	O2^xiii^—Ge1—O1^xiv^	92.73 (18)
O2^i^—Li1—Mo1^i^	46.7 (4)	O2—Ge1—O1^xv^	92.73 (17)
O2^ii^—Li1—Mo1^i^	46.7 (4)	O2^xii^—Ge1—O1^xv^	92.73 (17)
O2^iii^—Li1—Mo1^i^	96.7 (8)	O2^xiii^—Ge1—O1^xv^	166.8 (2)
O3—Li1—Mo1^iii^	140.4 (3)	O1^xiv^—Ge1—O1^xv^	77.0 (2)
O2^i^—Li1—Mo1^iii^	46.7 (4)	O2—Ge1—O1^xvi^	166.8 (2)
O2^ii^—Li1—Mo1^iii^	96.7 (8)	O2^xii^—Ge1—O1^xvi^	92.73 (17)
O2^iii^—Li1—Mo1^iii^	46.7 (4)	O2^xiii^—Ge1—O1^xvi^	92.73 (17)
Mo1^i^—Li1—Mo1^iii^	67.0 (4)	O1^xiv^—Ge1—O1^xvi^	77.0 (2)
O3—Li1—Mo1^ii^	140.4 (3)	O1^xv^—Ge1—O1^xvi^	77.0 (2)
O2^i^—Li1—Mo1^ii^	96.7 (8)	O4^xvii^—Mo1—O1^xvii^	104.58 (15)
O2^ii^—Li1—Mo1^ii^	46.7 (4)	O4^xvii^—Mo1—O1^viii^	104.58 (15)
O2^iii^—Li1—Mo1^ii^	46.7 (4)	O1^xvii^—Mo1—O1^viii^	76.0 (2)
Mo1^i^—Li1—Mo1^ii^	67.0 (4)	O4^xvii^—Mo1—O3	160.6 (3)
Mo1^iii^—Li1—Mo1^ii^	67.0 (4)	O1^xvii^—Mo1—O3	90.60 (16)
O3—Li1—Mo1^iv^	34.6 (2)	O1^viii^—Mo1—O3	90.60 (16)
O2^i^—Li1—Mo1^iv^	133.2 (4)	O4^xvii^—Mo1—O2	87.53 (16)
O2^ii^—Li1—Mo1^iv^	133.2 (4)	O1^xvii^—Mo1—O2	167.40 (14)
O2^iii^—Li1—Mo1^iv^	88.2 (3)	O1^viii^—Mo1—O2	97.8 (2)
Mo1^i^—Li1—Mo1^iv^	175.1 (5)	O3—Mo1—O2	78.36 (18)
Mo1^iii^—Li1—Mo1^iv^	116.94 (6)	O4^xvii^—Mo1—O2^v^	87.53 (16)
Mo1^ii^—Li1—Mo1^iv^	116.94 (6)	O1^xvii^—Mo1—O2^v^	97.8 (2)
O3—Li1—Mo1^v^	34.6 (2)	O1^viii^—Mo1—O2^v^	167.40 (14)
O2^i^—Li1—Mo1^v^	133.2 (4)	O3—Mo1—O2^v^	78.36 (18)
O2^ii^—Li1—Mo1^v^	88.2 (3)	O2—Mo1—O2^v^	86.0 (3)
O2^iii^—Li1—Mo1^v^	133.2 (4)	O4^xvii^—Mo1—Mo1^xviii^	51.91 (12)
Mo1^i^—Li1—Mo1^v^	116.94 (6)	O1^xvii^—Mo1—Mo1^xviii^	52.67 (9)
Mo1^iii^—Li1—Mo1^v^	175.1 (5)	O1^viii^—Mo1—Mo1^xviii^	90.54 (10)
Mo1^ii^—Li1—Mo1^v^	116.94 (6)	O3—Mo1—Mo1^xviii^	141.60 (11)
Mo1^iv^—Li1—Mo1^v^	59.0 (4)	O2—Mo1—Mo1^xviii^	139.30 (11)
O3—Li1—Mo1	34.6 (2)	O2^v^—Mo1—Mo1^xviii^	94.37 (14)
O2^i^—Li1—Mo1	88.2 (3)	O4^xvii^—Mo1—Mo1^xix^	51.91 (12)
O2^ii^—Li1—Mo1	133.2 (4)	O1^xvii^—Mo1—Mo1^xix^	90.54 (9)
O2^iii^—Li1—Mo1	133.2 (4)	O1^viii^—Mo1—Mo1^xix^	52.67 (9)
Mo1^i^—Li1—Mo1	116.94 (6)	O3—Mo1—Mo1^xix^	141.60 (11)
Mo1^iii^—Li1—Mo1	116.94 (6)	O2—Mo1—Mo1^xix^	94.37 (13)
Mo1^ii^—Li1—Mo1	175.1 (5)	O2^v^—Mo1—Mo1^xix^	139.30 (11)
Mo1^iv^—Li1—Mo1	59.0 (4)	Mo1^xviii^—Mo1—Mo1^xix^	60.0
Mo1^v^—Li1—Mo1	59.0 (4)	O4^xvii^—Mo1—Li1^xx^	85.0 (3)
O3—Li1—Li2	74.6 (5)	O1^xvii^—Mo1—Li1^xx^	139.97 (14)
O2^i^—Li1—Li2	74.9 (3)	O1^viii^—Mo1—Li1^xx^	139.97 (15)
O2^ii^—Li1—Li2	162.6 (10)	O3—Mo1—Li1^xx^	75.6 (3)
O2^iii^—Li1—Li2	74.9 (3)	O2—Mo1—Li1^xx^	43.02 (16)
Mo1^i^—Li1—Li2	120.8 (5)	O2^v^—Mo1—Li1^xx^	43.02 (16)
Mo1^iii^—Li1—Li2	65.8 (4)	Mo1^xviii^—Mo1—Li1^xx^	123.5 (2)
Mo1^ii^—Li1—Li2	120.8 (5)	Mo1^xix^—Mo1—Li1^xx^	123.5 (2)
Mo1^iv^—Li1—Li2	60.5 (4)	O4^xvii^—Mo1—Li1	169.2 (3)
Mo1^v^—Li1—Li2	109.2 (7)	O1^xvii^—Mo1—Li1	67.23 (18)
Mo1—Li1—Li2	60.5 (4)	O1^viii^—Mo1—Li1	67.23 (18)
O3—Li1—Li2^vi^	74.6 (5)	O3—Mo1—Li1	30.2 (3)
O2^i^—Li1—Li2^vi^	162.6 (10)	O2—Mo1—Li1	100.31 (19)
O2^ii^—Li1—Li2^vi^	74.9 (3)	O2^v^—Mo1—Li1	100.31 (19)
O2^iii^—Li1—Li2^vi^	74.9 (3)	Mo1^xviii^—Mo1—Li1	119.49 (18)
Mo1^i^—Li1—Li2^vi^	120.8 (5)	Mo1^xix^—Mo1—Li1	119.49 (18)
Mo1^iii^—Li1—Li2^vi^	120.8 (5)	Li1^xx^—Mo1—Li1	105.78 (5)
Mo1^ii^—Li1—Li2^vi^	65.8 (4)	Li2^xxi^—O1—Ge1^xxii^	124.9 (8)
Mo1^iv^—Li1—Li2^vi^	60.5 (4)	Li2^xxi^—O1—Mo1^xxiii^	119.4 (6)
Mo1^v^—Li1—Li2^vi^	60.5 (4)	Ge1^xxii^—O1—Mo1^xxiii^	103.46 (15)
Mo1—Li1—Li2^vi^	109.2 (7)	Li2^xxi^—O1—Mo1^xxiv^	119.4 (6)
Li2—Li1—Li2^vi^	113.2 (5)	Ge1^xxii^—O1—Mo1^xxiv^	103.46 (15)
O4—Li2—O1^vii^	101.1 (7)	Mo1^xxiii^—O1—Mo1^xxiv^	74.66 (17)
O4—Li2—O1^viii^	101.1 (7)	Ge1—O2—Li1^xx^	116.3 (6)
O1^vii^—Li2—O1^viii^	116.4 (5)	Ge1—O2—Mo1	125.62 (15)
O4—Li2—O1^ix^	101.1 (7)	Li1^xx^—O2—Mo1	90.3 (4)
O1^vii^—Li2—O1^ix^	116.4 (5)	Ge1—O2—Mo1^iv^	125.62 (15)
O1^viii^—Li2—O1^ix^	116.4 (5)	Li1^xx^—O2—Mo1^iv^	90.3 (4)
O4—Li2—Li1	74.6 (5)	Mo1—O2—Mo1^iv^	98.6 (2)
O1^vii^—Li2—Li1	65.0 (2)	Li1—O3—Mo1^iv^	115.18 (19)
O1^viii^—Li2—Li1	65.0 (2)	Li1—O3—Mo1	115.18 (19)
O1^ix^—Li2—Li1	175.6 (12)	Mo1^iv^—O3—Mo1	103.2 (2)
O4—Li2—Li1^x^	74.6 (5)	Li1—O3—Mo1^v^	115.18 (19)
O1^vii^—Li2—Li1^x^	175.6 (12)	Mo1^iv^—O3—Mo1^v^	103.2 (2)
O1^viii^—Li2—Li1^x^	65.0 (2)	Mo1—O3—Mo1^v^	103.2 (2)
O1^ix^—Li2—Li1^x^	65.0 (2)	Li2—O4—Mo1^iii^	134.58 (16)
Li1—Li2—Li1^x^	113.2 (5)	Li2—O4—Mo1^xxiv^	134.58 (16)
O4—Li2—Li1^xi^	74.6 (5)	Mo1^iii^—O4—Mo1^xxiv^	76.2 (2)
O1^vii^—Li2—Li1^xi^	65.0 (2)	Li2—O4—Mo1^xxiii^	134.58 (16)
O1^viii^—Li2—Li1^xi^	175.6 (12)	Mo1^iii^—O4—Mo1^xxiii^	76.2 (2)
O1^ix^—Li2—Li1^xi^	65.0 (2)	Mo1^xxiv^—O4—Mo1^xxiii^	76.2 (2)
Li1—Li2—Li1^xi^	113.2 (5)		

Symmetry codes: (i) *x*−*y*+1, *x*, *z*+1/2; (ii) −*x*+2, −*y*+2, *z*+1/2; (iii) *y*, −*x*+*y*+1, *z*+1/2; (iv) −*x*+*y*+1, −*x*+2, *z*; (v) −*y*+2, *x*−*y*+1, *z*; (vi) *x*−1, *y*, *z*; (vii) *x*−*y*, *x*−1, *z*−1/2; (viii) *y*+1, −*x*+*y*+2, *z*−1/2; (ix) −*x*+3, −*y*+1, *z*−1/2; (x) *x*+1, *y*, *z*; (xi) *x*, *y*−1, *z*; (xii) −*x*+*y*+2, −*x*+2, *z*; (xiii) −*y*+2, *x*−*y*, *z*; (xiv) −*x*+*y*+2, −*x*+2, *z*−1; (xv) −*y*+2, *x*−*y*, *z*−1; (xvi) *x*, *y*, *z*−1; (xvii) −*x*+3, −*y*+2, *z*−1/2; (xviii) −*x*+*y*+2, −*x*+3, *z*; (xix) −*y*+3, *x*−*y*+1, *z*; (xx) −*x*+2, −*y*+2, *z*−1/2; (xxi) −*x*+3, −*y*+1, *z*+1/2; (xxii) *x*, *y*, *z*+1; (xxiii) *x*−*y*+1, *x*−1, *z*+1/2; (xxiv) −*x*+3, −*y*+2, *z*+1/2.
